# Brigatinib can inhibit proliferation and induce apoptosis of human immortalized keratinocyte cells

**DOI:** 10.3389/fphar.2025.1524277

**Published:** 2025-02-18

**Authors:** Qi Yang, Dan Zhao, Linjie Ju, Peng Cao, Jifu Wei, Zhixian Liu

**Affiliations:** ^1^ School of Pharmacy, Nanjing University of Chinese Medicine, Nanjing, China; ^2^ Jiangsu Cancer Hospital, Jiangsu Institute of Cancer Research, The Affiliated Cancer Hospital of Nanjing Medical University, Nanjing, China

**Keywords:** brigatinib, amphiregulin, epiregulin, TGFA, PI3K/AKT

## Abstract

**Background:**

Brigatinib is approved in multiple countries for the treatment of patients with anaplastic lymphoma kinase (ALK)-positive non-small cell lung cancer (NSCLC). Despite its superior efficacy, the dermal toxicities caused by brigatinib cannot be overlooked. However, its underlying mechanism remains unknown.

**Methods:**

The effects of brigatinib on the proliferation ability of human immortalized keratinocyte (HaCaT) cells were evaluated using Cell Counting Kit-8 (CCK-8) proliferation, colony formation, and 5-ethynyl-2′-deoxyuridine (EdU) incorporation assays. The effects of brigatinib on apoptosis were detected using Annexin FITC/PI and Acridine Orange (AO) staining assays. Cell cycle was assessed with flow cytometry. An analysis of transcriptome by RNA sequencing procedures (RNA-seq) was performed to reveal the key regulatory genes. Gene Ontology (GO) and Kyoto Encyclopedia of Genes and Genomes (KEGG) were used to find out the biological function and related signal pathways. The expressions of amphiregulin, epiregulin and transforming growth factor alpha (TGFA) and the protein levels of Phosphoinositide 3-kinase (PI3K)/protein kinase B (AKT) and Cleaved-Caspase three were measured by quantitative reverse transcription polymerase chain reaction (qRT-PCR) and western blot assay.

**Results:**

Brigatinib inhibits cell proliferation with an IC_50_ value of 2.9 μmol/L and significantly increases apoptosis rates. Transcriptome sequencing (RNA-seq) indicates that brigatinib could significantly downregulate the expression of amphiregulin, epiregulin and TGFA. In addition, we demonstrated that brigatinib reduced the protein expression of amphiregulin, epiregulin, TGFA, PI3K, AKT and phosphorylated AKT (p-AKT).

**Conclusion:**

This study confirms the inhibition of HaCaT cells growth and progression by brigatinib and highlights the potential value of the PI3K/AKT pathway as a therapeutic target for brigatinib-induced dermal toxicities.

## 1 Introduction

Non-small cell lung cancer (NSCLC) globally comprises approximately 85% of lung cancer, which stands as the primary cause of cancer-related mortality (Russell et al., 2022). In general, current therapies for NSCLC include surgical treatments, chemotherapy, radiotherapy and targeted therapy (Guo et al., 2022). Although surgical removal of the primary tumor has beneficial effects, the physiological stress caused by surgical trauma can promote cancer recurrence and metastasis (Market et al., 2021). Radiotherapy uses ionizing radiation to target and destroy tumor tissue. But it can damage normal tissue, leading to severe toxicity (De Ruysscher et al., 2019). Nevertheless, the effectiveness of traditional chemotherapy treatments is usually hindered by the toxic side effects and tumor heterogeneity (Huo et al., 2024). Nowadays, targeted therapy has become a crucial alternative to manage NSCLC due to its higher efficacy and fewer side effects. Among all the promising targeted drugs, brigatinib, one of the second-generation anaplastic lymphoma kinase (ALK) tyrosine kinase inhibitors (ALK-TKIs), has robust therapeutic efficacy in patients with ALK-positive NSCLC compared with crizotinib (Camidge et al., 2018).

Brigatinib received approval by the US Food and Drug Administration (FDA) in 2017 and by the European Medicines Agency (EMA) in 2018. In China, brigatnib was approved for the first-line treatment option of patients diagnosed with ALK-positive locally advanced or metastatic NSCLC by the marketing approval from the China National Food and Drug Administration on 24 March 2022.

Unfortunately, severe dermal toxicities sometimes force the patients to prematurely stop their treatments. Research on 136 patients who received brigatinib treatment indicated that almost 45% of them suffered from negative dermal toxicities, including pruritus (18%), rashes (15%), acne-like dermatitis (9%), and red rashes (3%) (Camidge et al., 2020). Despite its remarkable effectiveness in treatment, the dermal toxicities resulting from brigatinib cannot be disregarded. These dermal toxicities often lead to the early discontinuation of brigatinib (Lichtenberger et al., 2013). Previous researches have confirmed some possible mechanisms among dermal toxicities caused by ALK-TKIs and the immune system (Gleue et al., 2021). However, the molecular mechanisms of brigatinib-induced dermal toxicities are not well-understood. Therefore, it is crucial to gain insight into the underlying mechanisms of dermal toxicities associated with brigatinib in order to enhance the efficacy of anti-ALK–based cancer therapies and mitigate the adverse effects on patients.

In view of the previous reports, we knew that epidermal growth factor receptor (EGFR) inhibitors induce dermal toxicities by the blockade of the receptor tyrosine kinase signaling pathway, such as PI3K/AKT pathway (Campbell et al., 2014; Kudo et al., 2018; Satoh et al., 2020). Some authors believed that vascular endothelial growth factor receptor (VEGFR) inhibitors could hypothetically have an effect on the vascular repair mechanisms in the body, leading to a marked inflammation after any kind of vascular damage, including skin inflammation (Lee et al., 2009; Ishak et al., 2014). V-raf murine sarcoma viral oncogene homolog B1 (BRAF) inhibitors-induced dermal toxicities are common because the of paradoxical activation of the mitogen-activated protein kinase (MAPK) pathway in keratinocyte cells (Heidorn et al., 2010; Bhargava et al., 2016; Bancalari et al., 2019). However, it remains to be elucidated how brigatinib affects skin cells.

Amphiregulin has the ability to either promote or suppress the growth of different normal and cancer cell lines (Johnson et al., 1991). Stoll *et al* demonstrated that amphiregulin can induce keratinocyte cells proliferation (Stoll et al., 2010). Epiregulin plays a role in cutaneous excisional wound healing by promoting angiogenesis (Roy et al., 2008). Transforming growth factor alpha (TGFA) modulates several cellular processes including, differentiation, growth and apoptosis (Gaba and Jain, 2024). In this study, we discovered that brigatinib significantly downregulated the expressions of amphiregulin, epiregulin and TGFA in skin cells.

In the present study, we firstly design an *in vitro* stimulation of human immortalized keratinocyte (HaCaT) cells by brigatinib to explore its effect on keratinocyte cells proliferation and apoptosis. Our study sheds light on several promising therapeutic target for releasing the dermal toxicities induced by brigatinib.

## 2 Materials and methods

### 2.1 Reagents

Brigatinib (HY-12857) was purchased from MedChemExpress (MCE, Shanghai, China) with the purity of 99.98%. The relevant materials used in this study were provided as following: Acridine Orange staining detection kit (AO, KGA 1811-100, KeyGEN, Nanjing, China), PI/RnaseA cell cycle detection kit (KGA9101-100, KeyGEN, Nanjing, China), Annexin V-FITC/PI Staining kit (KGA107, KeyGEN, Nanjing, China), Crystal violet (P0013B, Beyotime, Shanghai, China), Trizol (P0016, Beyotime, Shanghai, China), Phosphate buffered saline (PBS, KGL2206-500, KeyGEN, Nanjing, China), RIPA (P00138, Beyotime, Shanghai, China), BeyoClick™ EdU-555 cell proliferation detection kit (C0075S, Beyotime, Shanghai, China), Cell Counting Kit-8 Kit (CCK-8, E1CK-000208, EnoGene, Nanjing, China), FreeZol reagent (R711-01, Vazyme, Nanjing, China), AceQ Universal SYBR qPCR master mix (Q511-02, Vazyme, Nanjing, China), Evo M-MLV RT mix kit (AG11728, Accurate, Changsha, China), Enhanced chemiluminescence detection reagent (ECL, 180–501, Tanon, Shanghai, China).

The antibodies used in Western Blotting assay were obtained as following: PI3K (1:600, 20584-1-AP, Proteintech, Wuhan, China), AKT (1:5000, 60203-2-AP, Proteintech, Wuhan, China), p-Ak (1:2000, 4060T, Cell signaling technology, United States), Amphiregulin (1:1000, 16036-1-AP, Proteintech, Wuhan, China), Epiregulin (1:1000, PK94578S, abmart, Shanghai, China), TGFA (1:1000, T58262S, abmart, Shanghai, China), Cleaved-Caspase 3 (1:1000, YM3431, Immunoway, Suzhou, China), GAPDH (1:60000, 60004-1-AP, Proteintech, Wuhan, China), HRP-conjugated Affinipure Goat Anti-Rabbit IgG (1:5000, SA00001-2, Proteintech, Wuhan, China), HRP-conjugated Affinipure Goat Anti-Mouse IgG (1:5000, SA00001-1, Proteintech, Wuhan, China).

### 2.2 Cell culture

The human immortalized keratinocyte cells HaCaT were purchased from Shanghai Fuheng Biotechnology Co., Ltd. HaCaT cells were cultured in Dulbecco’s modified Eagle’s medium (DMEM, KGL1202-500, KeyGEN, Nanjing, China) supplemented with 10% fetal bovine serum (FBS, A6901FBS, Invigentech, United States) and 1% penicillin and streptomycin (C0222, Beyotime, Shanghai, China) with 5% CO_2_ at 37°C.

### 2.3 Cells model establishment

To establish brigatinib activated cells, HaCaT cells were treated with brigatinib for 48 h at the concentrations of 0, 3.125, 6.25, 12.5, 25, 50, and 100 μmol/L, respectively. To explore whether brigatinib influences the cellular proliferation ability and apoptosis, we employed brigatinib at 2.9 μmol/L to theHaCaT cells for 48 h.

### 2.4 CCK-8 assay

HaCaT cells were seeded in 96-well plate (1 × 10^4^cells/well) and treated with brigatinib at different concentration for another 48 h. After incubation, 10 μL CCK-8 solution was added into each well, and the 96-well plate was subsequently incubated at 37°C for 2 h in the dark. The optical density value (OD) was detected at a wavelength of 450 nm to determine the cells viability by a SpectraMax-iD5 multifunctional microplate reader (Molecular Devices, Shanghai, China). The half maximal inhibitory concentrations (IC_50_) values were calculated using the GraphPad Prism software (v.9.0).

### 2.5 Colony formation assay

HaCaT cells were seeded in six-well plate (500 cells/well). After 24 h of incubation, the cells were treated with brigatinib for another 48 h. Then washed by PBS and cultured in fresh medium for 10 days. The fresh medium was replaced every 3 days. Cells were fixed with 4% paraformaldehyde solution for 30 min and stained with crystal violet solution for 20 min. Finally, cells were washed several times with PBS and captured by a camera. Colony numbers were manually counted through ImageJ software. A colony containing more than 50 cells was counted as one colony.

### 2.6 EdU incorporation assay

HaCaT cells were seeded in six-well plate (2 × 10^4^ cells/well) and treated with brigatinib for 48 h. BeyoClick™ EdU-555 cell proliferation detection kit was used for the subsequent experiments. Briefly, 20 μmol/L EdU was added into per well for 2 h. Hoechst 33342 was used to stain the nuclei for 10 min in the dark. The images collected with 20×visions in fluorescence microscope (Zeiss, Germany).

### 2.7 Acridine Orange (AO) staining method

HaCaT cells were seeded in 24-well plates at a density of 2 × 10^4^ cells/well and treated with brigatinib for 48 h. Then, AO staining solution was added under light-proof conditions and incubated in the incubator for 15 min. The cell morphology was observed under a fluorescent inverted microscope with 40×visions (Zeiss, Germany).

### 2.8 Cell apoptosis analysis

In the cell apoptosis assay, Annexin V-FITC/PI apoptosis detection kit was applied according to the manufacturer’s instructions. HaCaT cells were seeded in six-well plate (2 × 10^4^ cells/well). After treated with brigatinib for 48 h, cells were collected and sequentially stained with Annexin V-FITC and PI solution for 10 min. The cells were analyzed by flow cytometry (BD Biosciences, United States). Data were analyzed by Flowjo software (v.10.8.1).

### 2.9 Cell cycle analysis

HaCaT cells were seeded in six-well plate (5 × 10^4^ cells/well). After treated with brigatinib for 48 h, harvested and washed twice with PBS, and then fixed in cold ethanol (70%). The cells were stained with propidium iodide (20 μg/mL) and RNase A (0.2 mg/mL) for 60 min. The stained cells were analyzed by flow cytometry (BD Biosciences, United States) and the data were analyzed with Modfit software (v.5.0).

### 2.10 RNA sequencing procedures (RNA-seq)

HaCaT cells were treated with brigatinib. After treatment for 48 h, the total RNA was extracted by Trizol regent and sequenced in Beijing Biomarker Technology Co., LTD. (Beijing, China). Then the prepared RNA-seq libraries were sequenced by an Illumina Novaseq 6,000 sequencer (Biomarker Technologies, Beijing, China). RNA concentration and purity were measured using NanoDrop 2000 (Thermo Fisher Scientific, Wilmington, DE). RNA integrity was assessed using the RNA Nano 6000 Assay Kit of the Agilent Bioanalyzer 2,100 system (Agilent Technologies, CA, United States). Differential expression analysis of two groups was performed using the DESeq2. The resulting *P* values were adjusted using the Benjamini and Hochberg’s approach for controlling the false discovery rate. Genes with an adjusted *P*-value <0.01 & Fold Change≥2 found by DESeq2 were assigned as differentially expressed. Volcano plot was generated using GraphPad Prism (v.9.0).

### 2.11 Gene ontology (GO) and yoto encyclopedia of genes and genomes (KEGG) analysis

GO enrichment analysis of the differentially expressed genes (DEGs) was implemented by the clusterProfiler packages based Wallenius non-central hyper-geometric distribution, which can adjust for gene length bias in DEGs. We used KOBA database and clusterProfiler software to test the statistical enrichment of differential expression genes in KEGG pathways. The Hiplot Pro (https://hiplot.com.cn/) was utilized to visualize.

### 2.12 Quantitative reverse transcription polymerase chain reaction (qRT-PCR)

HaCaT cells were seeded in six-well plate (2 × 10^5^ cells/well). After treated with brigatinib for 48 h, total cellular RNA was extracted using Freezol reagent and cDNA was synthesized using Evo M-MLV Mix Kit with gDNA Clean. Quantitative PCR (qPCR) was performed using AceQ Universal SYBR qPCR Master Mix and was run on the QuantStudio™ 6 Flex real-time PCR system. GAPDH was set as the control gene for normalization. Three independent biological replications were performed for each experiment. The relative fold-change was calculated by the 2^-△△Ct^ method. Primers used in this study were synthesized by Invitrogen Biotechnology Co., Ltd. (Invitrogen, United States) and gene-specific primers were listed in Supplementary Table S1.

### 2.13 Western blotting

Total proteins from the HaCat cells in 6-well plates were extracted using RIPA lysis buffer with 2% Protease and phosphatase inhibitor. The extracted proteins were separated using 10% SDS electrophoresis before transfer onto a PVDF membrane. The membranes were blocked with 5% non-fat milk at 37°C for 1 h and were incubated with primary antibodies at 4°C overnight. Secondary antibodies pre-labeled in room temperature for 1 h. ECL detection system (Tanon, Shanghai, China) was employed to detect immunoreactive bands. Protein band intensities were analyzed by ImageJ software.

### 2.14 Statistical analyses

Statistical analyses were acquired with GraphPad Prism (v.9.0). Data were presented as mean ± SD. The one-way analysis of variance (ANOVA), the Student’s t-test were carried out for comparison between groups. All the experiments were repeated three times. *P*-value <0.05 was statistically significant.

## 3 Results

### 3.1 Brigatinib could inhibit the growth and proliferation of HaCaT cells

**FIGURE 1:**
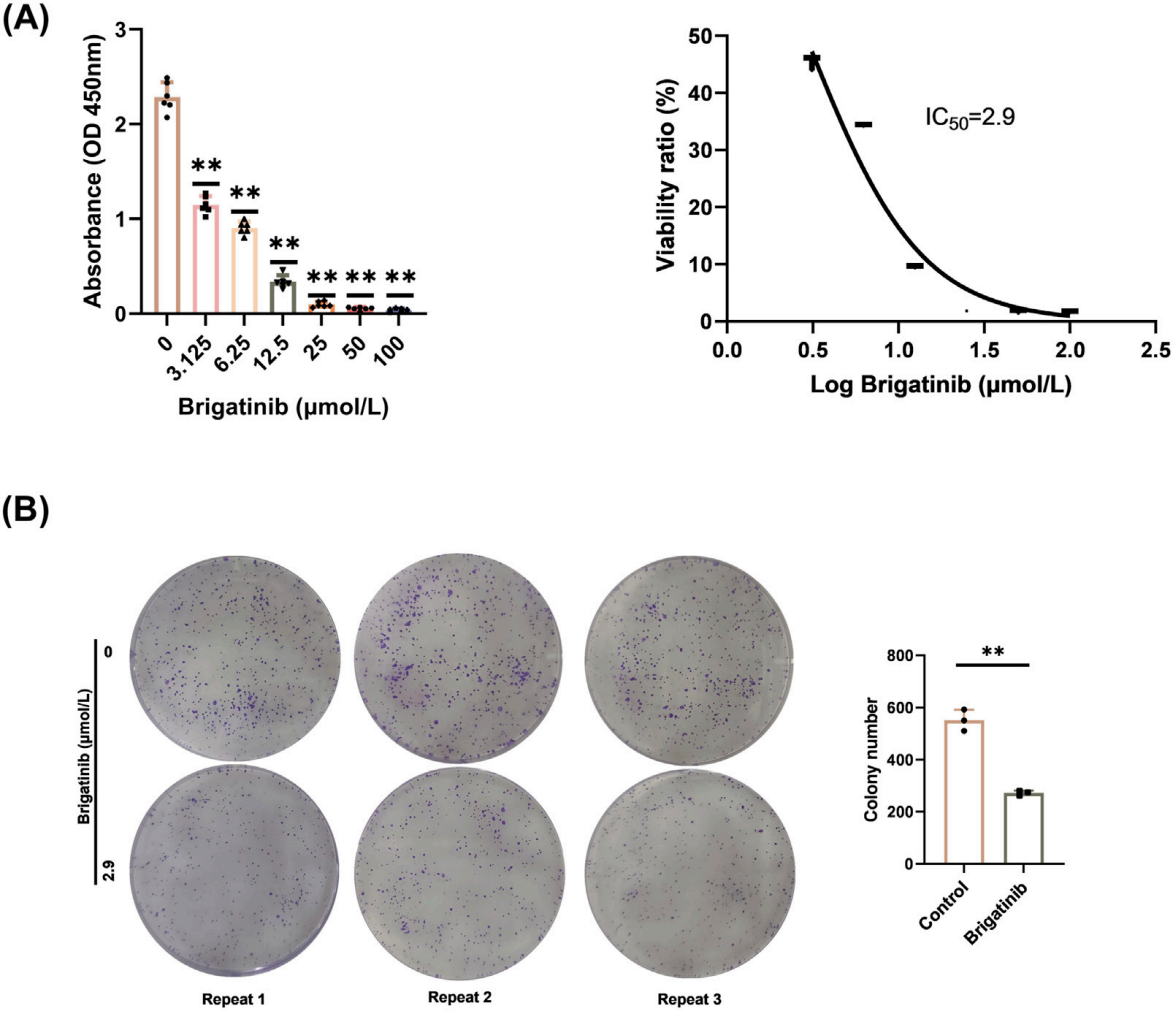
Brigatinib could dramatically inhibit the growth and proliferation of HaCaT cells. **(A)** CCK8 assay was performed to identify the growth rate of HaCaT cells. Data are shown as mean ± SD, n = 6 (^*^ *P <* 0.05, ^**^ *P <* 0.01) **(B)** Brigatinib decreased the colony-forming ability of the HaCaT cell. Data are shown as mean ± SD, n = 3 (^**^ *P <* 0.01).

In order to explore the effect of brigatinib treatment on HaCaT cells proliferation, CCK8 assay was performed to identify the growth rate of HaCaT cells. The brigatinib concentration gradient ranged from 3.125 μmol/L to 100 μmol/L. As shown in Figure 1A, brigatinib significantly inhibited the growth of HaCaT cells in a dose-dependent manner with IC_50_ as 2.9 μmol/L after 48 h of treatment. Drug toxicity refers to the harmful effects of long-term or high-dose use of drugs on the body (Edwards and Aronson, 2000). Zhang et al. (2016) found that brigatinib potently inhibited ALK activity and proliferation in all ALK^+^ cell lines at IC_50_ ranged from 4 to 31 nmol/L. Our study found that brigatinib inhibited HaCaT cells proliferation at 2.9 μmol/L, which indicated that brigatinib has a significant cytotoxic effect on HaCaT cells during long-term or high-dose treatment. Then, we chose 2.9 μmol/L as the optimal concentration of brigatinib for the further cell colony formation experiment. As shown in Figure 1B, the colony-forming ability of the HaCaT cells was significantly inhibited after brigatinib treatment. In conclusion, brigatinib could dramatically inhibit the growth and proliferation of HaCaT cells.

### 3.2 Brigatinib could induces the apoptosis of HaCaT cells

**FIGURE 2:**
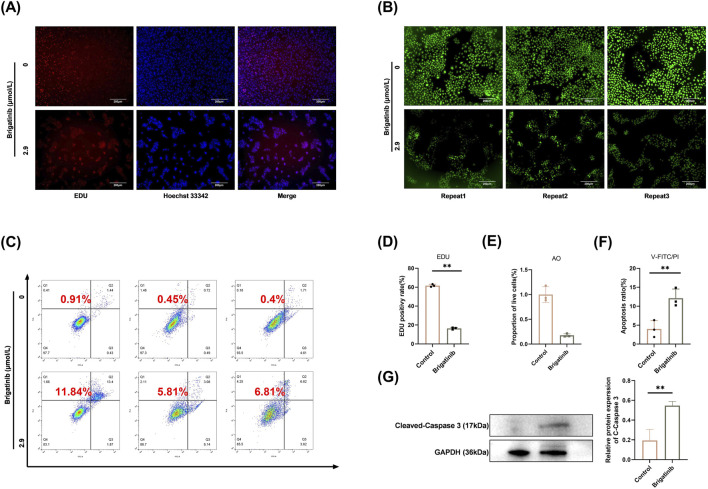
Brigatinib could induce the apoptosis of HaCaT cells. **(A)** Decreased number of EdU positive HaCaT cells was shown after brigatinib treatment. **(B)** Fluorescence of apoptosis induced by brigatinib on HaCaT cells using AO staining. **(C)** Brigatinib could significantly increase the proportion of apoptotic HaCaT cells. **(D)** EdU positivy rate of HaCaT cells. Data are shown as mean ± SD, n = 3 (^**^ *P <* 0.01) **(E)** Proportion of live cells in AO staining. Data are shown as mean ± SD, n = 3 (**P < 0.01) **(F)** Apoptosis ratio of HaCaT cells. Data are shown as mean ± SD, n = 3 (^**^ *P <* 0.01) **(G)** Western blots analysis of Cleaved-Caspase three in HaCaT cells after brigatinib treatment. Data are shown as mean ± SD, n = 3 (**P < 0.01).

To explore the impact of brigatinib on HaCaT cells apoptosis, we employed EdU incorporation and AO staining assays. EdU incorporation assay showed a significant reduction in the number of EdU-positive cells following brigatinib treatment (Figures 2A, D; Supplementary Figure S1), suggesting that entrectinib could inhibit the replicative capacity of HaCaT cells. AO staining can distinguish between normal cells and apoptotic cells. Under the microscope, the green fluorescence formed by the control group was evenly distributed. While at the concentration of 2.9 μmol/L, it was clearly observed that fluorescence by the cells decreased significantly, which meant that the number of apoptosis cells increased (Figures 2B, E). We used flow cytometry apoptotic analysis to further investigate the induced apoptosis effect of brigatinib on HaCaT cells. We found that brigatinib could significantly increase the proportion of apoptotic HaCaT cells (Figures 2C, F). Moreover, we then assessed apoptosis by performing western blot analysis for apoptotic marker. As shown in Figure 2G; Supplementary Figure S2, the protein level of Cleaved-Caspase three was enhanced. These results suggest that brigatinib could induce the apoptosis of HaCaT cells significantly.

### 3.3 Brigatinib could suppress HaCaT cells proliferation via G1/S phase cell cycle arrest

**FIGURE 3:**
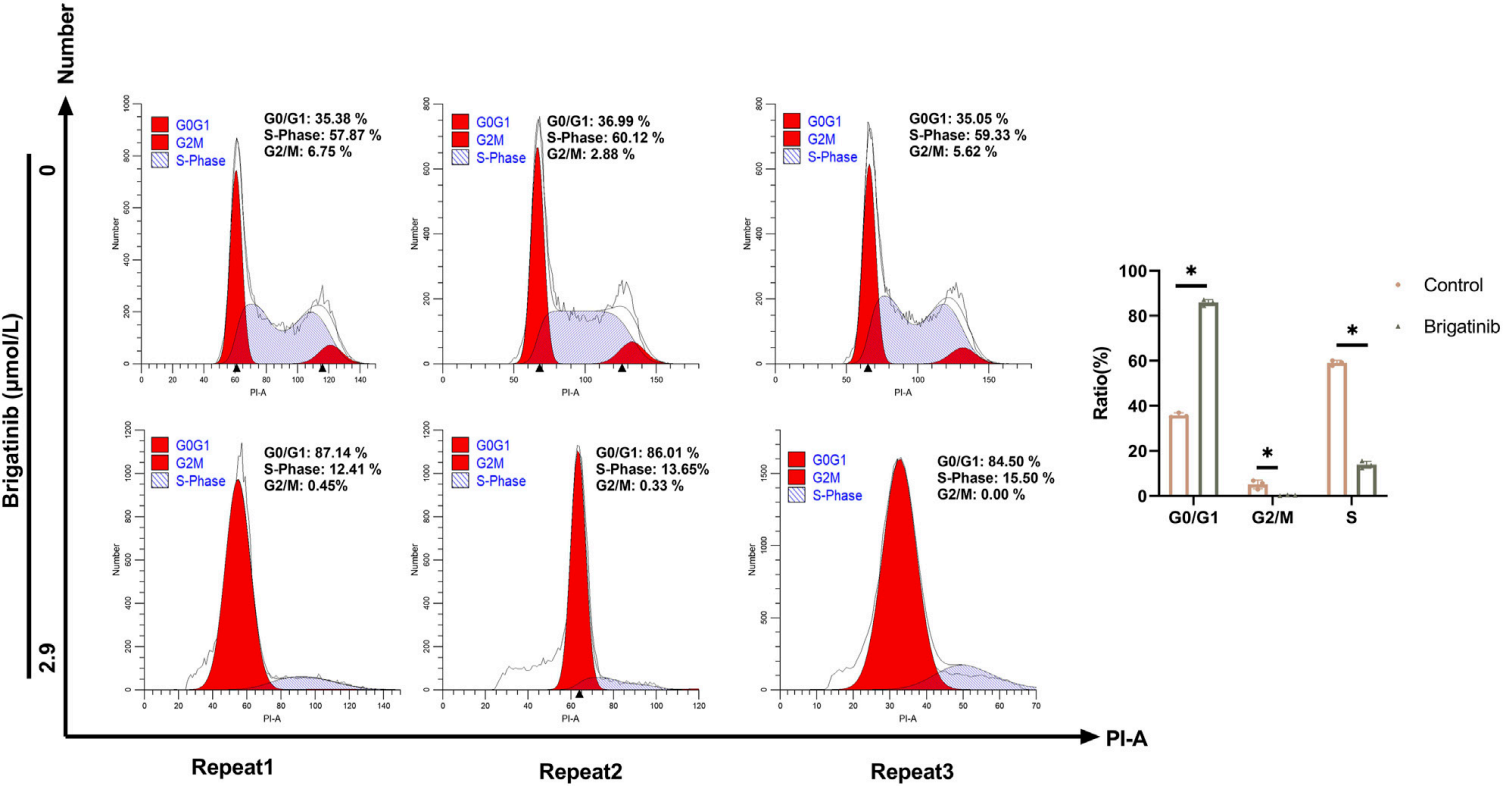
Brigatinib could induced G1/S phase cell cycle arrest in HaCaT cells. The proportions of cells in the G0/G1, G2/M, and S phases were shown. Data are shown as mean ± SD, n = 3 (^*^ *P <* 0.05).

To ensure the specific cell cycle blocked by brigatinib, flow cytometry was utilized to detect the cell cycle of HaCaT cells incubated by brigatinib. As shown in Figure 3, the G0/G1 phase percentage of HaCaT cells was significantly increased compared with the control group. Moreover, a significant decrease in S phase percentage was detected after brigatinib treatment. The above results indicated that brigatinib could induce G1/S cell cycle arrest in HaCaT cells.

### 3.4 GO analysis of differentially expressed genes after brigatinib treatment in HaCaT cells

**FIGURE 4:**
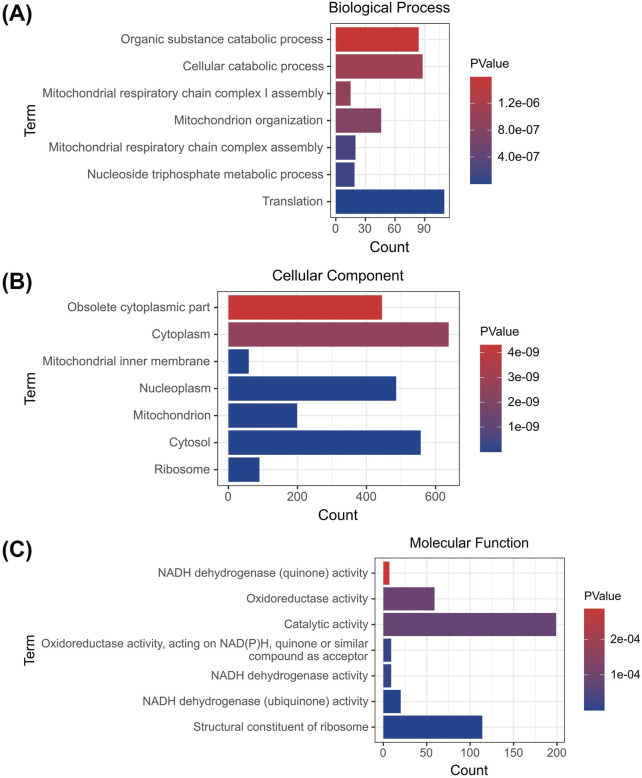
GO analysis of DEGs after brigatinib treatment in HaCaT cells. **(A)** GO- biological process analysis. **(B)** GO-cellular component analysis. **(C)** GO-molecular function analysis.

GO analysis of the HaCaT cells of DEGs were conducted to predict DEGs functions and molecular interactions among genes after brigatinib treatment. GO analyses covered three domains: biological process, cellular component and molecular function. The top seven enriched GO terms in biological process, cellular component and molecular function after brigatinib treatment were shown in Figures 4A–C and Supplementary Table S2. Significantly enriched GO terms for biological processes included organic substance catabolic process, cellular catabolic process and mitochondrial respiratory chain complex I assembly. In terms of cellular component, obsolete cytoplasmic part, cytoplasm and mitochondrial inner membrane were significantly enriched. Finally, for the molecular functions category, NADH dehydrogenase (quinone) activity, oxidoreductase activity and catalytic activity were significantly enriched after brigatinib treatment.

### 3.5 KEGG pathway analysis of differentially expressed genes after brigatinib treatment in HaCaT cells

**FIGURE 5:**
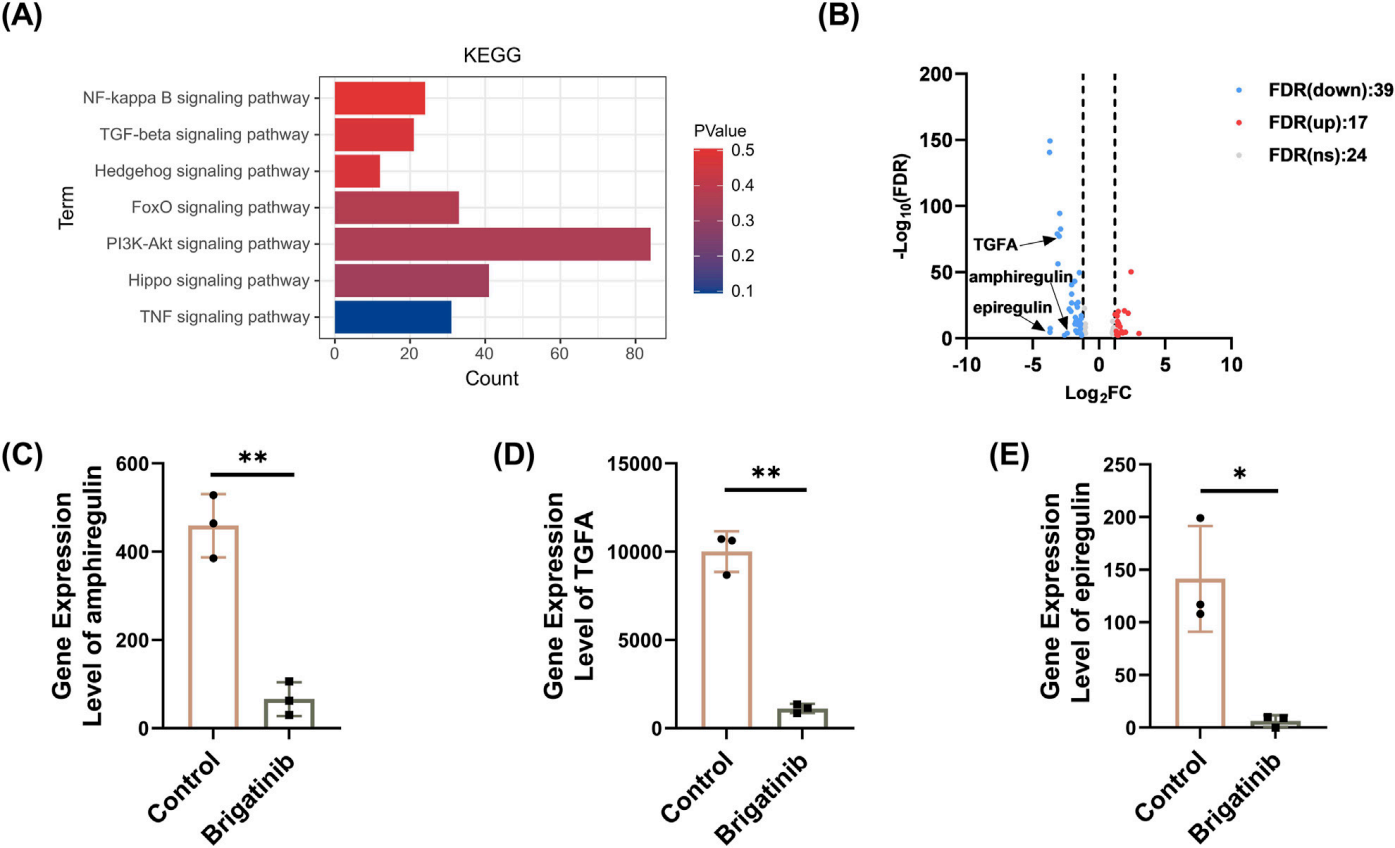
KEGG pathway analysis of differentially expressed genes after brigatinib treatment in HaCaT cells. **(A)** Top 7 KEGG pathways of KEGG enrichment analysis in HaCaT cells after brigatinib treatment. **(B)** The volcano plot of DEGs in PI3K/AKT pathway after brigatinib treatment in HaCaT cells, where red represents 17 upregulated genes, blue represents 39 downregulated genes, and gray represents 24 genes with insignificant differences (Set threshold FDR <0.05 and **|**fold change**|** > 2) **(C–E)** Gene differential expression analysis of amphiregulin, epiregulin, and TGFA in HaCaT cells after brigatinib treatment. Data are shown as mean ± SD, n = 3 (^*^ *P <* 0.05, ^**^ *P <* 0.01).

KEGG analysis of DEGs was performed to find out the related signal pathway in HaCaT cells after brigatinib treatment. The results of KEGG pathway analysis are were presented in Figure 5A; Supplementary Table S3. There was a significant enrichment of DEGs in gene sets related to the PI3K/AKT signaling pathway. As the PI3K/AKT pathway is a crucial signaling pathway in cellular processes such as proliferation and apoptosis, the 57 target genes in the PI3K/AKT pathway were analyzed in detail (Figure 5B; Supplementary Table S4). Among this pathway, 18 genes were downregulated and 39 genes were upregulated in HaCaT cells after brigatinib treatment. To further explore the functions of apoptotic, we focused on amphiregulin, epiregulin and transforming growth factor alpha (TGFA), which were significantly downregulated after brigatinib stimulation (Figures 5C–E).

### 3.6 Brigatinib could downregulate amphiregulin, epiregulin and TGFA expressions and inhibit the PI3K/AKT signaling pathway of HaCaT cells

**FIGURE 6:**
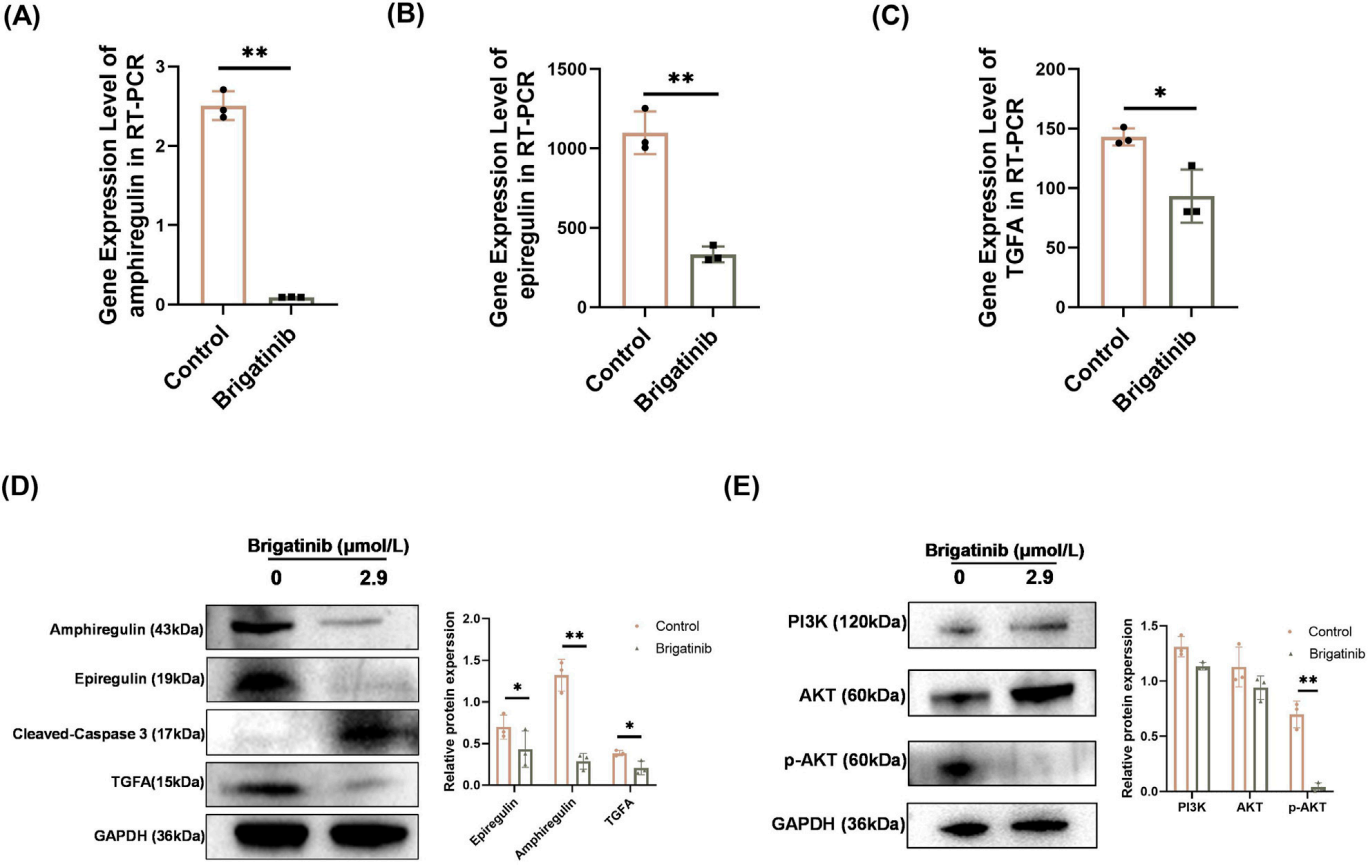
Brigatinib inhibited the PI3K/AKT pathway of HaCaT cells and decreasing amphiregulin, epiregulin, and TGFA expressions. **(A–C)** qRT-PCR analysis of amphiregulin, epiregulin, and TGFA in HaCaT cells after brigatinib treatment. Data are shown as mean ± SD, n = 3 (^*^ *P <* 0.05, ^**^ *P <* 0.01) **(D)** Western blots analysis of amphiregulin, epiregulin, TGFA in HaCaT cells after brigatinib treatment. Data are shown as mean ± SD, n = 3 (^*^ *P <* 0.05, ^**^ *P <* 0.01) **(E)** Western blots analysis of PI3K, AKT and p-AKT in HaCaT cells after brigatinib treatment. Data are shown as mean ± SD, n = 3 (^**^ *P <* 0.01).

Subsequently, experiments were conducted to validate whether brigatinib stimulation resulted in a reduction in the expression of associated target genes. Consistent with RNA-seq results, qRT-PCR (Figures 6A–C) and western blotting results (Figure 6D; Supplementary Figure S3-5) showed that the expressions of amphiregulin, epiregulin and TGFA were decreased. To investigate whether PI3K/AKT in HaCaT cells were inhibited by brigatinib, we examined the expression of PI3K, AKT, and p-AKT protein levels in HaCaT cells after brigatinib treatment. As illustrated in Figure 6E; Supplementary Figure S6, the protein level of p-AKT was dramatically decreased in HaCaT cells after brigatinib treatment. Taken together, these results demonstrated that brigatinib could downregulate the expressions of amphiregulin, epiregulin and TGFA and inhibit the PI3K/AKT pathway.

## 4 Discussion

According to the clinical practice guidelines issued by the National Comprehensive Cancer Network (NCCN), brigatinib was recommended as the first-line treatment option for the patients with ALK-positive advanced NSCLC (Benson et al., 2021). Nevertheless, the dermal toxicities including itching, rashes, acneiform dermatitis and erythema, etc., bring great pain to the patients and even become the main reason for drug discontinuation (Yoshida et al., 2023). Unfortunately, few investigations elucidate the mechanism of brigatinib-induced dermal toxicities.

Epithelia tissue within the skin act as the critical barriers in the body and require continual renewal to sustain barrier integrity. The skin epithelium plays a particularly crucial role against external pathogens and other environmental hazards. Hence, inhibition of skin cell proliferation can result in the disruption of the skin barrier, which may cause skin disorders. The prevailing view is that apoptosis is initiated by a regulated signaling pathway or by an unregulated process resulting from cellular damage (Golstein and Kroemer, 2007; Rothlin and Ghosh, 2020; Newton et al., 2024). Jiang *et al* found that lapatinib induced mitochondrial dysfunction, caused DNA damage, and ultimately resulted in apoptosis of HaCaT cells, which owing to the decreased High mobility group box 1 (HMGB1) expression (Jiang et al., 2022). Penny *et al* reported keratinocyte cells surface deposition of IgG and proliferation by direct immunofluorescence in patients treated with novel antibody-drug (Penny et al., 2022). Nanba *et al* found that EGFR suppression promoted type XVII collagen (COL17A1) proteolysis, which decreased the clonal growth of keratinocyte cells (Nanba et al., 2021). In our studies, brigatinib was also proved to inhibit HaCaT cells proliferation and promote its apoptosis. Our findings were in parallel with the previous reports that drug rashes were related to the significant increase apoptosis rate of HaCaT cells (Mlacki et al., 2014; Zhuang et al., 2021).

We conducted further analysis to elucidate the alterations in transcriptional levels of HaCaT cells upon brigatinib treatment though RNA-seq analysis. We noticed that the expressions of amphiregulin, epiregulin, and TGFA were significantly downregulated followed by brigatinib treatment. Amphiregulin, epiregulin, and TGFA are the upstream signals of PI3K/AKT (Xu et al., 2016; Martin et al., 2017; Mohseni et al., 2021), which are the members of the EGF family and have different abilities to activate EGF receptors (Graus-Porta et al., 1997; Maretzky et al., 2011). According to qRT-PCR validation, we demonstrated that the results were strongly consistent with the RNA-seq data. amphiregulin associated with physiological processes, especially proliferation of keratinocyte proliferation (Cook et al., 1991). Schelfhout *et al* demonstrated that amphiregulin could restore the tissue integrity following infection or injury (Schelfhout et al., 2002). Epiregulin is also involved in wound healing, inflammation and cell proliferation in skin. Shirasawa *et al* suggested that loss of epiregulin could cause chronic dermatitis in mice and releases key molecules of pro-inflammatory factors (Stoll et al., 2001; Shirasawa et al., 2004). TGFA is a polypeptide structurally related to EGF and the second member of the EGF receptor ligand family identified after the discovery of the prototype member EGF. Singh *et al* indicated that TGFA knockout mice have significant early wound epithelial damage (Singh and Coffey, 2014). Likewise, TGFA is also considered to be extensively involved in the wound healing process (Pandiella and Massague, 1991). Kim *et al* also reported that gefitinib-treated keratinocytes were observed slight decrease of p-AKT and PI3K (Kim et al., 2020). In our study, we further validated the expressions of amphiregulin, epiregulin and TGFA of HaCaT cells after briagatinib treatment. The results indicated that the expressions of amphiregulin, epiregulin and TGFA were significantly downregulated by brigatinib, which were consistent with RNA-seq results.

Interestingly, the results of KEGG analysis revealed that the DEGs were involved in many important proliferation and apoptosis related pathways, such as PI3K/AKT pathway. PI3K is a heterodimer composed of catalytic subunits and regulatory subunits. When it binds to the appropriate target, it triggers a series of downstream reactions. AKT is a type of serine/threonine kinase, which can be activated by phosphorylation of PI3K-related kinase (PIKK) (Chaussade et al., 2007; Bozulic and Hemmings, 2009; Manning and Toker, 2017). The classical PI3K/AKT pathway is related to the regulation of a variety of physiological activities, including cell proliferation, differentiation, apoptosis, angiogenesis, metabolism, and protein synthesis (She et al., 2005; Porta et al., 2014). In skin, the activation of the PI3K/AKT pathway is responsible for maintaining the skin homeostasis (Westin, 2014). Peng *et al* demonstrated that AKT-deficient mice without cuticle will cause death of young mice (Peng et al., 2003). In addition, studies showed that the PI3K/AKT pathway is related to the occurrence and development of skin diseases such as psoriasis, atopic dermatitis, and vitiligo (Mitra et al., 2012). Similarly, we conducted Western blots to determine the level of PI3K/AKT. Consistent with our expectations, the level of p-AKT was markedly downregulated after brigatinib treatment. According to the previous report, inhibition of the activated EGFR in normal epithelial tissues results in inhibition of extracellular regulated protein kinases (ERK) 1/2 phosphorylation (pERK) and decreased keratinocyte proliferation and migration (Lacouture et al., 2021). Remarkably, brigatinib is also active in cell lines with mutations in the gene encoding EGFR (Katayama et al., 2011). Our findings preliminarily confirmed that brigatinib-induced dermal toxicities are mediated by the downregulation of amphiregulin, epiregulin, and TGFA and suppression of the PI3K/AKT pathway.

These findings not only elucidate the molecular mechanisms of brigatinib-induced dermal toxicities but also provide a basis for therapeutic strategies targeting the PI3K/AKT pathway.

## 5 Conclusion

**FIGURE 7:**
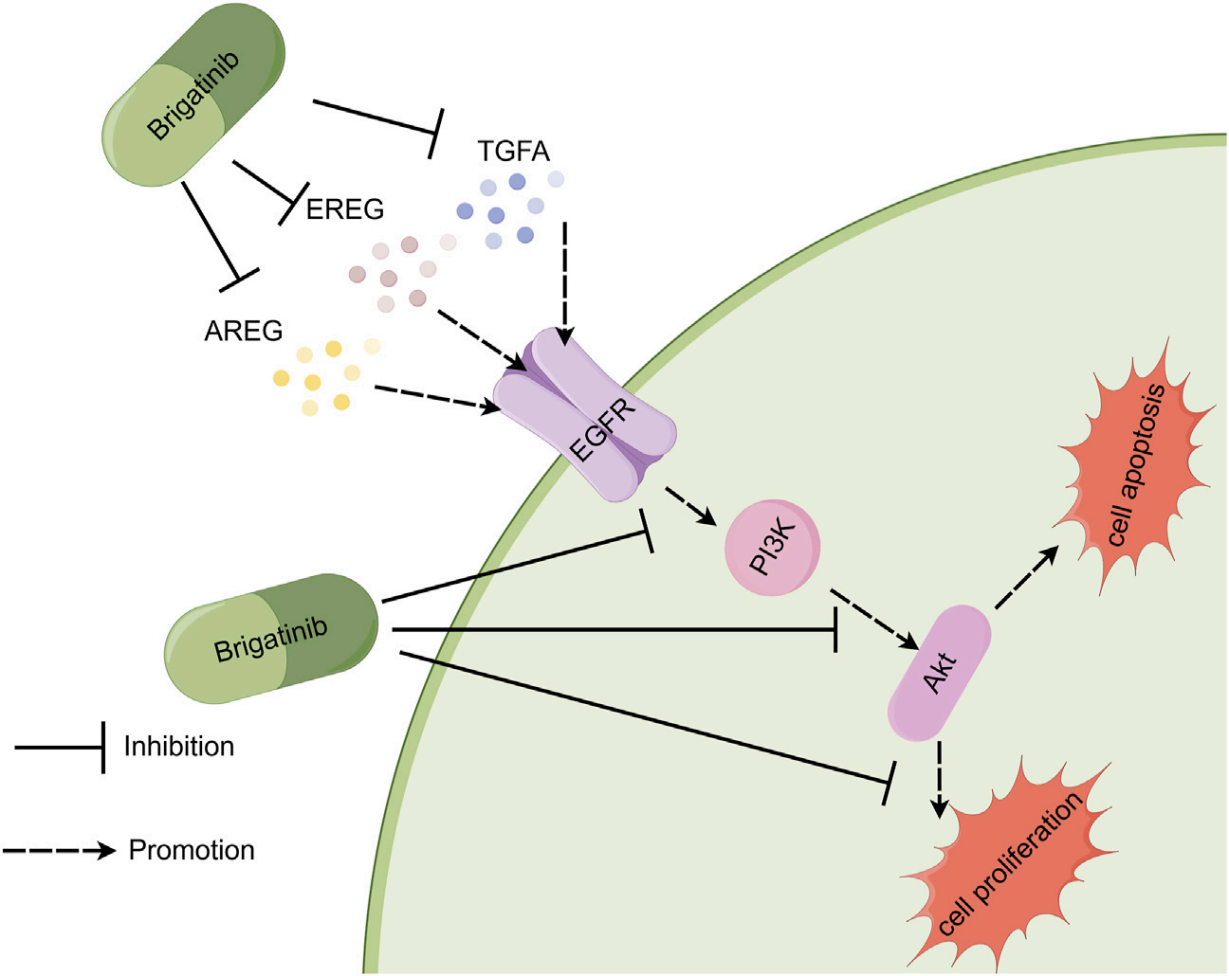
Proposed mechanism of brigatinib in inhibiting proliferation and promoting apoptosis on HaCaT cells. Brigatinib induced HaCaT cells damage by downregulating amphiregulin, epiregulin, and TGFA expressions while also inhibiting PI3K/AKT signaling pathways. The figure was drawn by Figdraw.

In essence, our findings indicated the inhibitory impact of brigatinib on amphiregulin, epiregulin, and TGFA expressions and a considerable decline in PI3K/AKT pathway activity in HaCaT cells (Figure 7). Our study suggests that targeting PI3K/AKT pathway may be therapeutic in brigatinib-induced dermal toxicities. These findings provided a theoretical basis for the development of clinical drugs for brigatinib-induced dermal toxicities.

## Data Availability

The data presented in the study are deposited in the Sequence Read Archive (SRA) repository, accession number PRJNA1220196.
