# Supplementary figures and images for: Brigatinib can inhibit proliferation and induce apoptosis of human immortalized keratinocyte cells
Source: Front Pharmacol. 2025 Feb 18;16:1524277. doi: 10.3389/fphar.2025.1524277 (PMC11876137; doi:10.3389/fphar.2025.1524277)

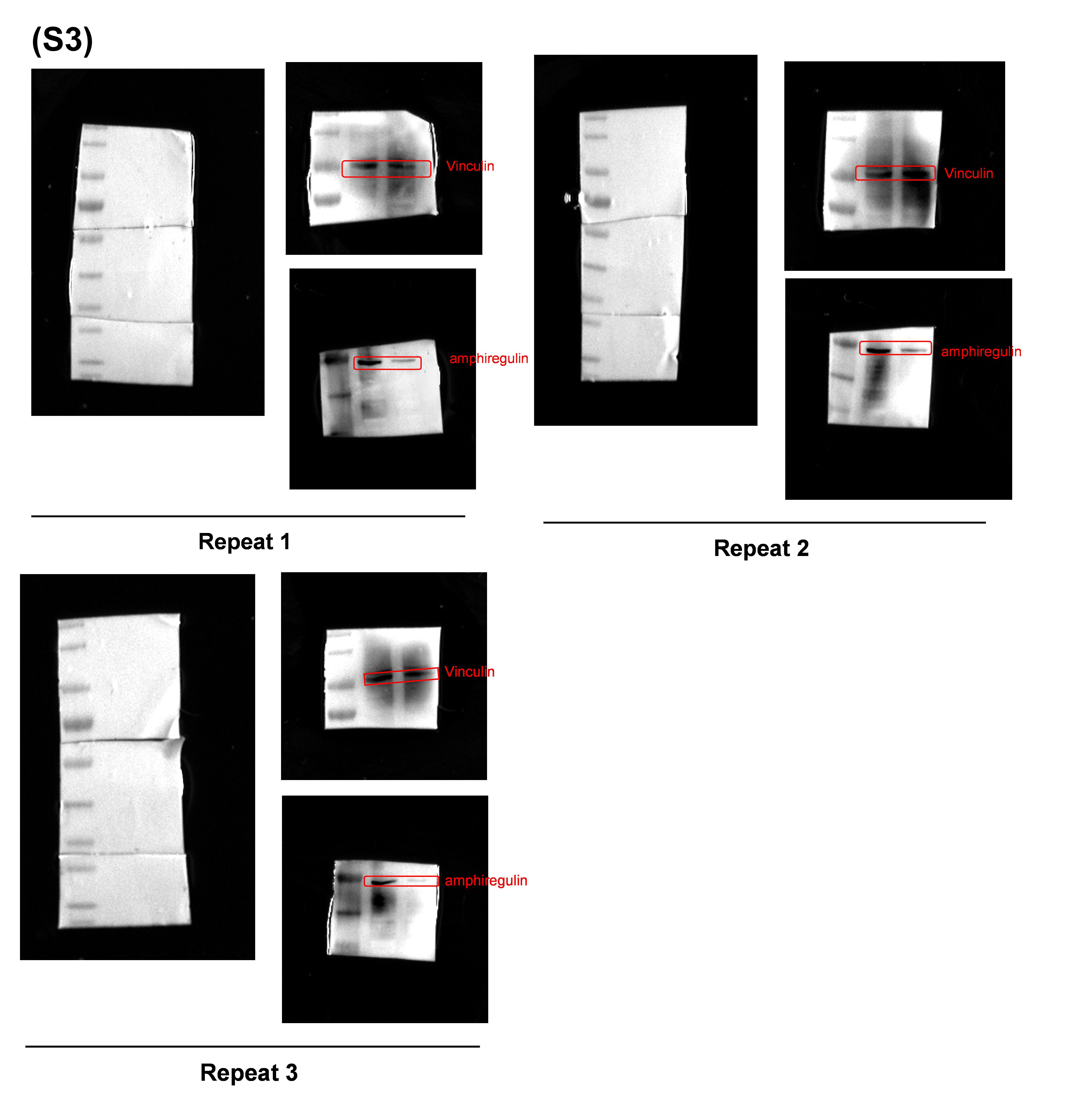

Supplement: Supplementary file 2 [file Image3.jpeg]

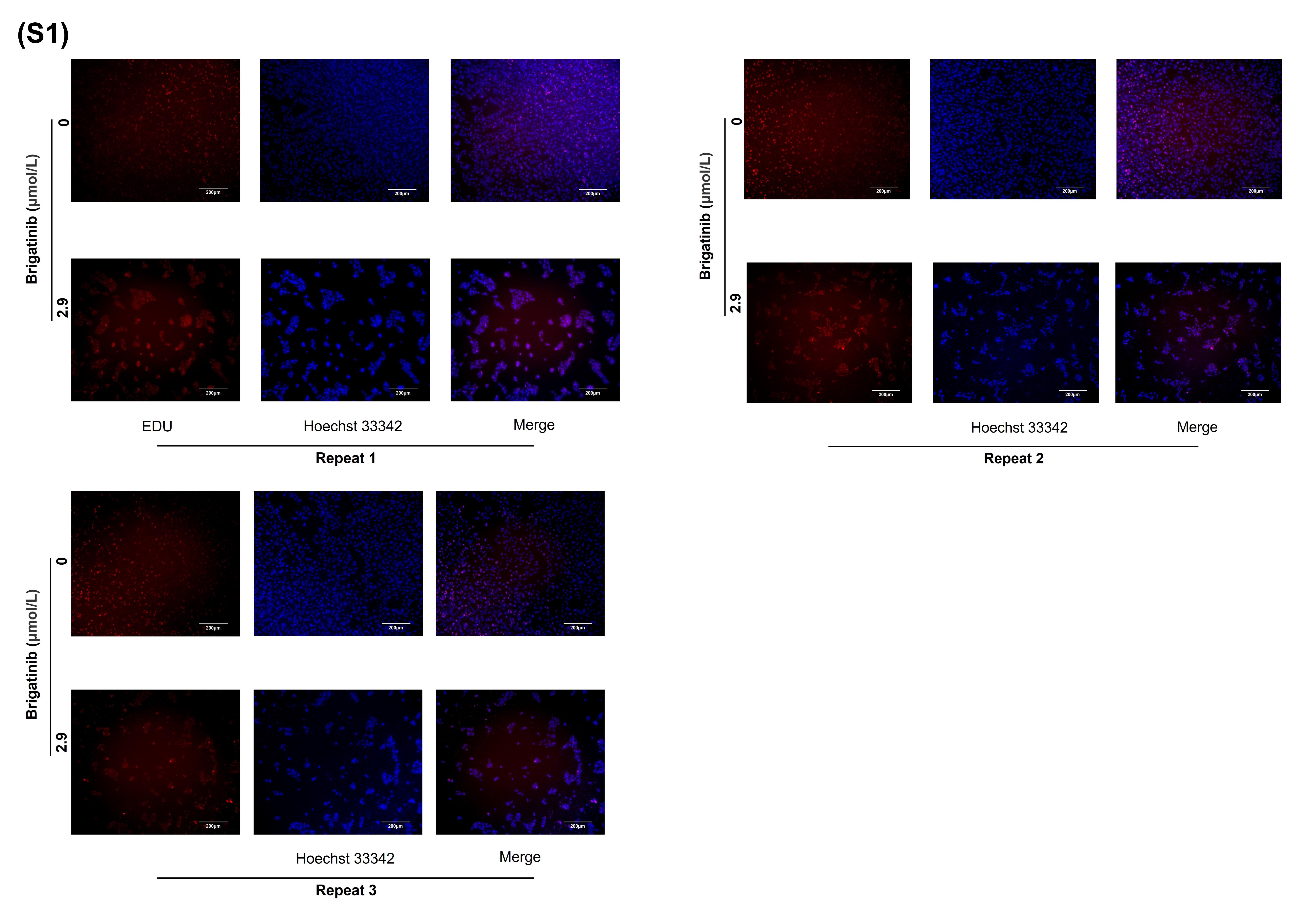

Supplement: Supplementary file 5 [file Image1.jpeg]

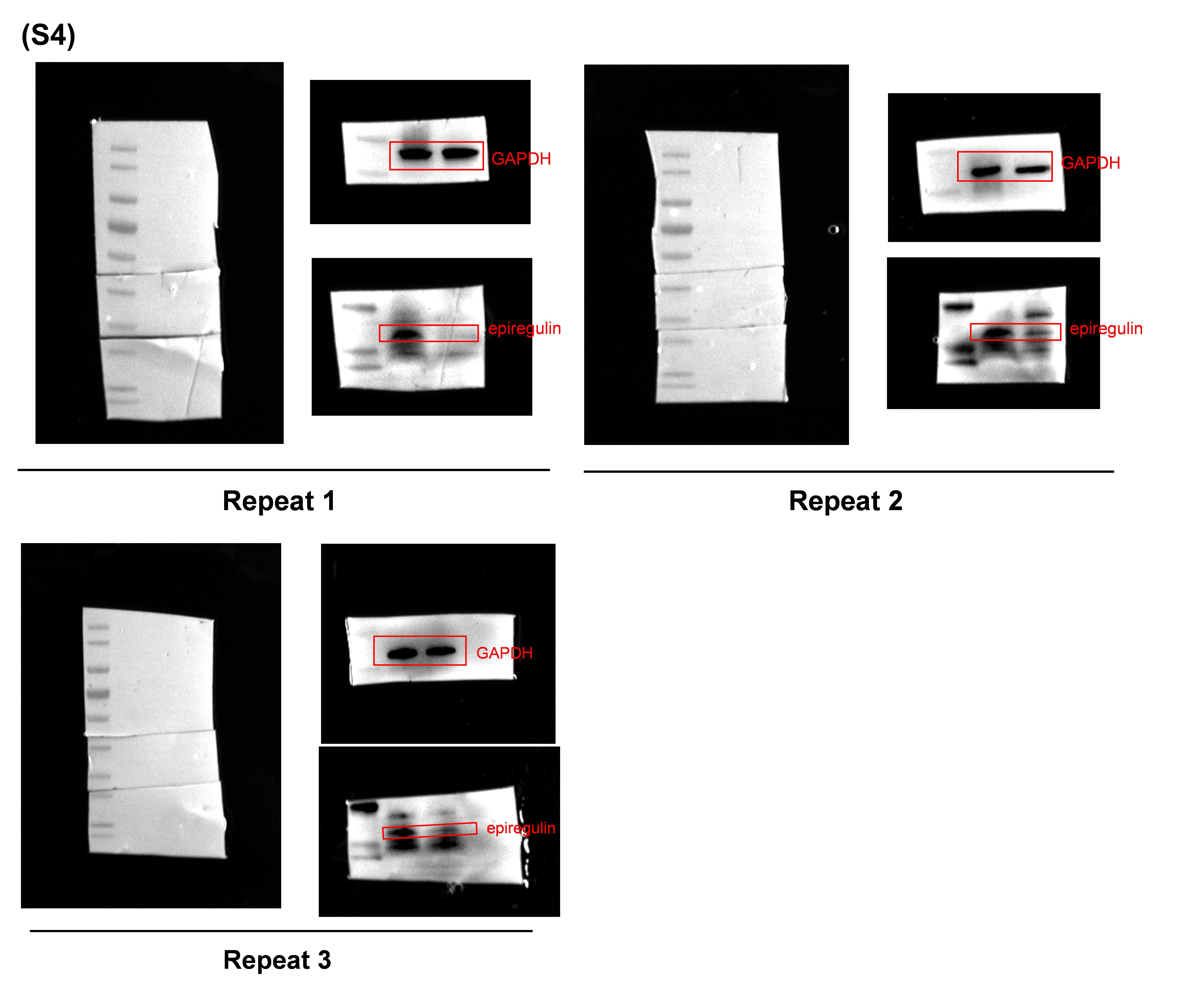

Supplement: Supplementary file 6 [file Image4.jpeg]

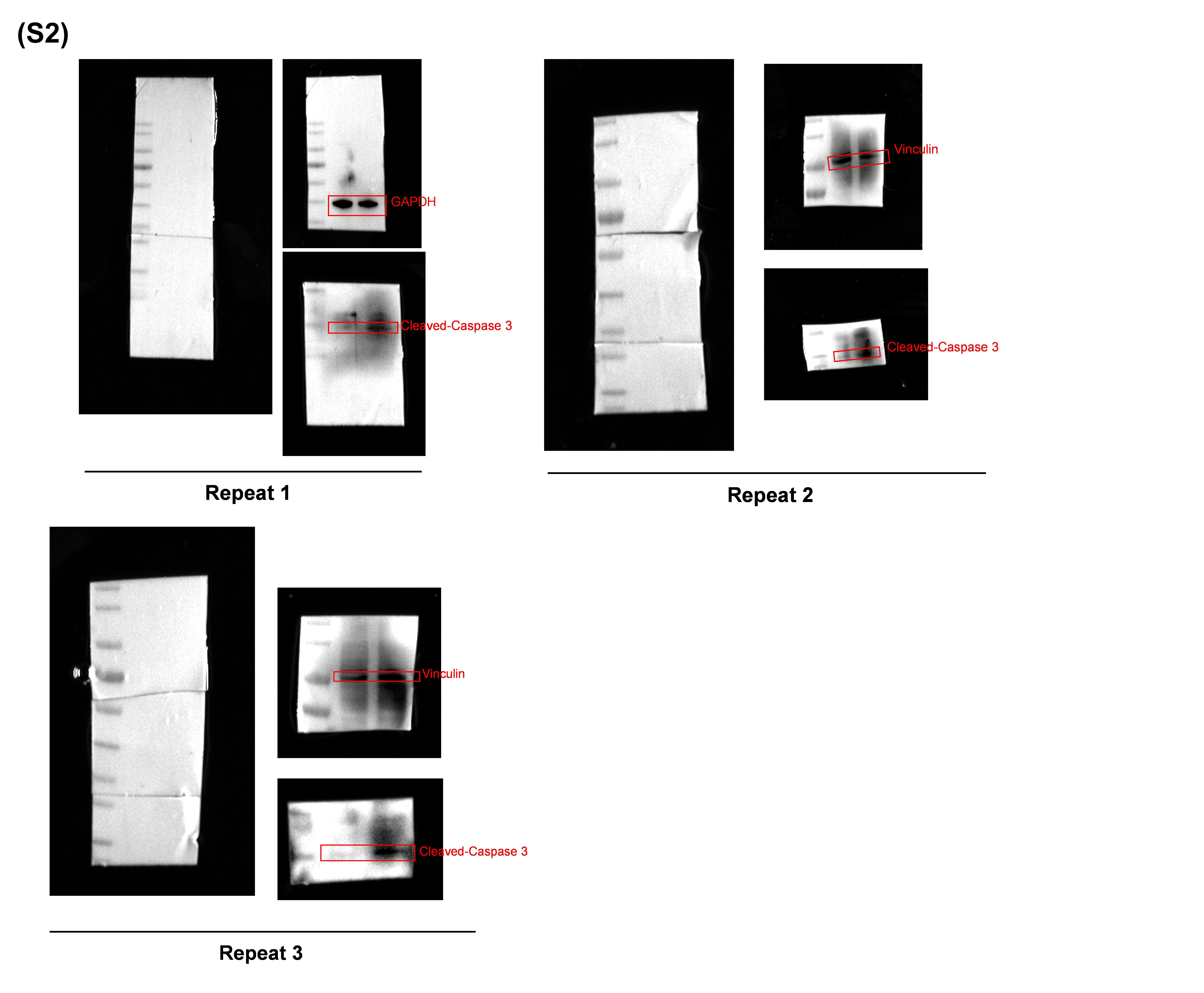

Supplement: Supplementary file 7 [file Image2.jpeg]

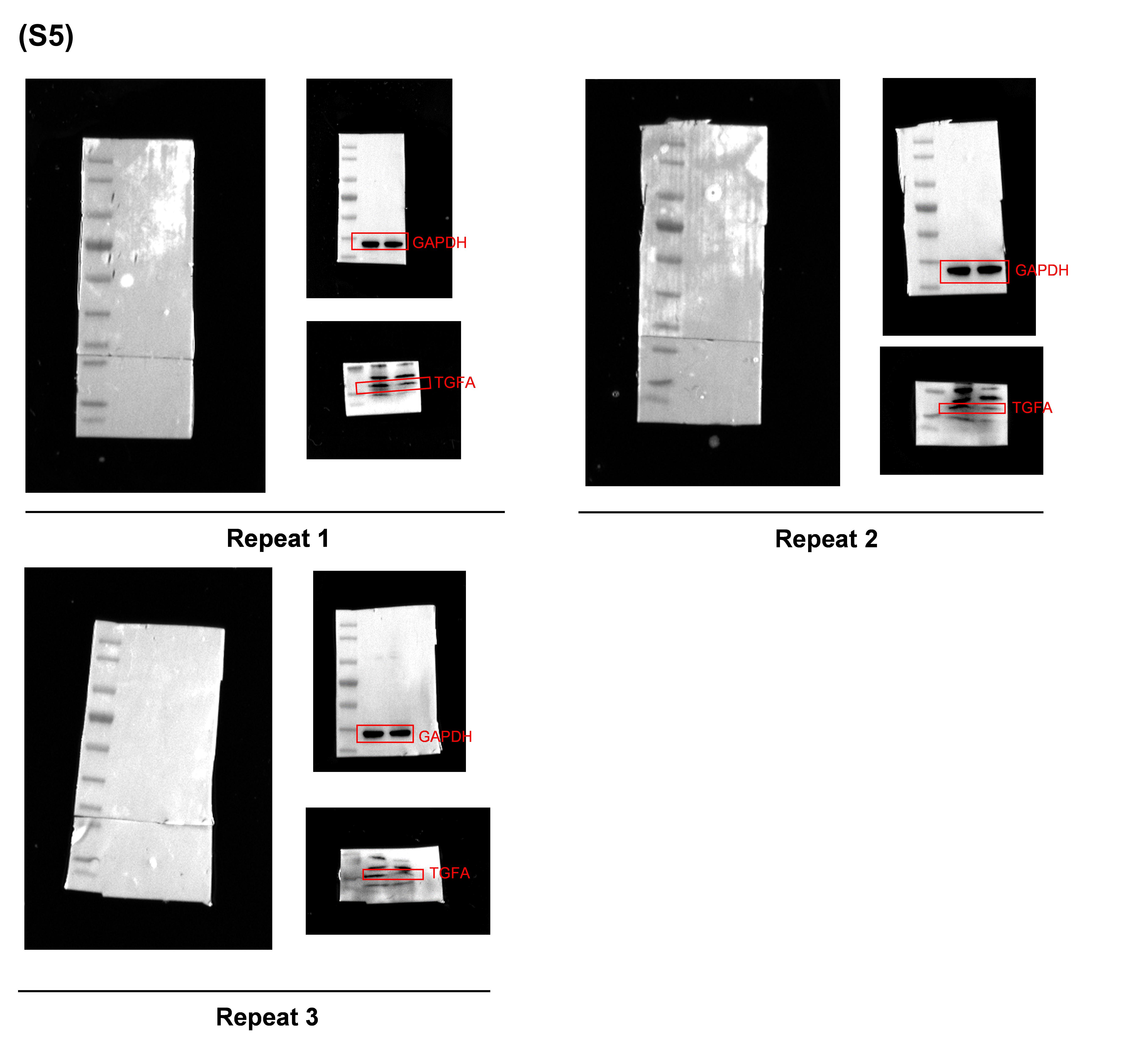

Supplement: Supplementary file 8 [file Image5.jpeg]

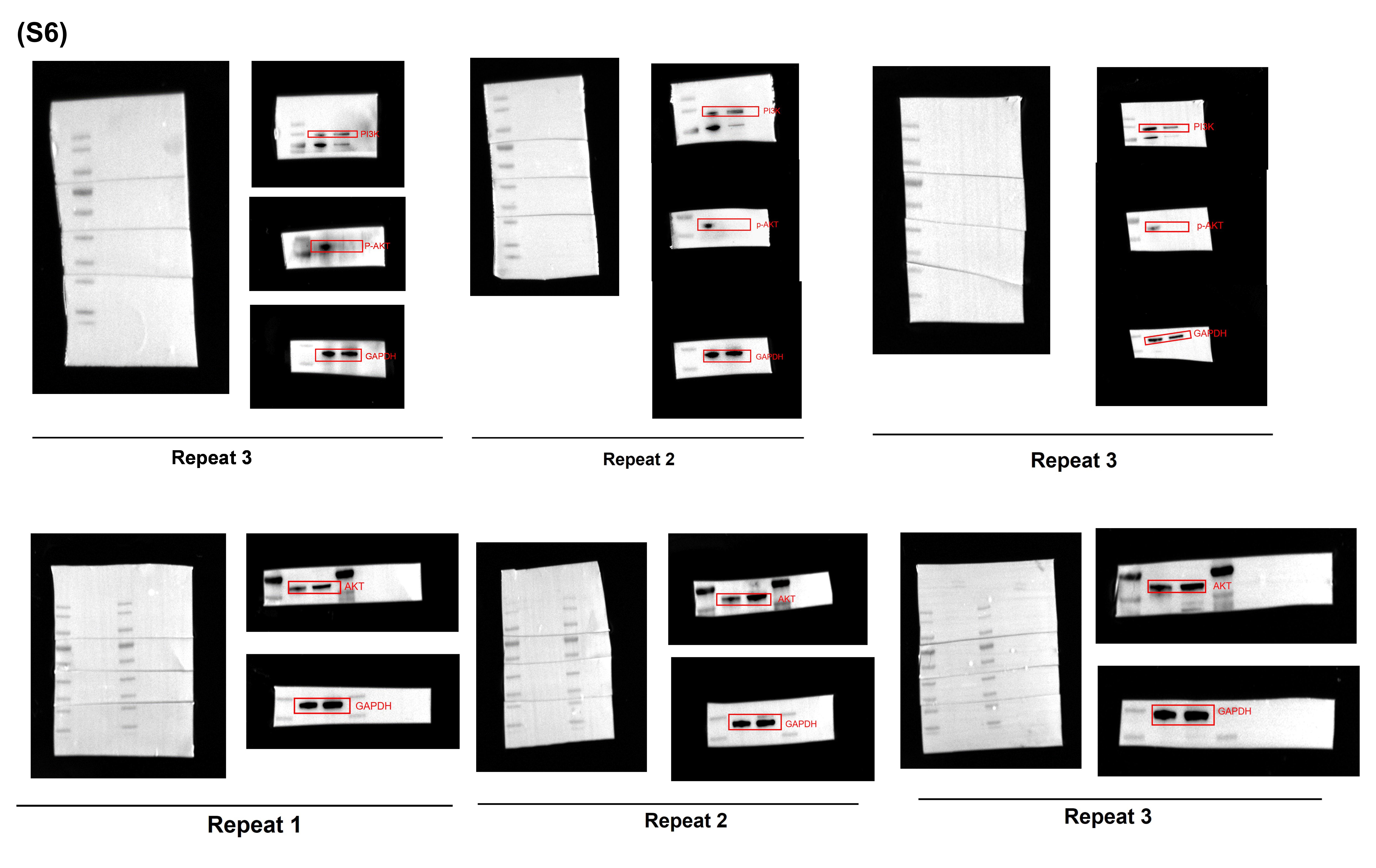

Supplement: Supplementary file 10 [file Image6.jpeg]
