# Supplementary material for: Brigatinib can inhibit proliferation and induce apoptosis of human immortalized keratinocyte cells
Source: Front Pharmacol. 2025 Feb 18;16:1524277. doi: 10.3389/fphar.2025.1524277 (PMC11876137; doi:10.3389/fphar.2025.1524277)
Supplement: Supplementary file 4 [file Table1.docx]

**Supplementary Table S1.** The qPCR primers used in this study

| **Primer name** | **Sequences (5’-3’)** | **Sequences (3’-5’)** |
| --- | --- | --- |
| Amphiregulin | GTGGTGCTGTCGCTCTTGATA | CCCCAGAAAATGGTTCACGCT |
| Epiregulin | GTGATTCCATCATGTATCCCAGG | GCCATTCATGTCAGAGCTACACT |
| TGFA | AGGTCCGAAAACACTGTGAGT | AGCAAGCGGTTCTTCCCTTC |
| GAPDH | GGAGCGAGATCCCTCCAAAAT | GGCTGTTGTCATACTTCTCATGG |
